# Stimulating *Anopheles gambiae* swarms in the laboratory: application for behavioural and fitness studies

**DOI:** 10.1186/s12936-015-0792-2

**Published:** 2015-07-15

**Authors:** Luca Facchinelli, Laura Valerio, Rosemary S Lees, Clelia F Oliva, Tania Persampieri, C Matilda Collins, Andrea Crisanti, Roberta Spaccapelo, Mark Q Benedict

**Affiliations:** Department of Experimental Medicine, University of Perugia, Perugia, Italy; Centre for Environmental Policy, Imperial College London, London, UK; Department of Life Sciences, Imperial College London, London, UK; Centers for Disease Control and Prevention, Atlanta, USA

**Keywords:** *Anopheles gambiae*, Swarms, Mating behaviour, Genetic control, Competitiveness

## Abstract

**Background:**

Male *Anopheles* mosquitoes that swarm rely in part on features of the environment including visual stimuli to locate swarms. Swarming is believed to be the primary behaviour during which mating occurs in the field, but is not a common behaviour in the laboratory. Features that stimulate male *Anopheles gambiae* G3 strain swarming were created in novel large indoor cages.

**Methods:**

The following visual features were tested in all combinations to determine which were important for swarm formation. Large cages and fading ceiling lights at dusk alone did not stimulate swarming while a dark foreground and contrasting illuminated background with a contrasting landmark stimulated and localized swarm formation during artificial twilight. Given the need to test transgenic strains in as natural a setting as possible, in this study it was investigated whether induced swarm behaviour and cage size would affect relative mating performance of wild-type and transgenic β2Ppo1 and β2Ppo2 *A.* *gambiae* sexually sterile males.

**Results:**

Even using a mosquito colony that has been in laboratory culture for 39 years, swarming behaviour was induced by this novel arrangement. The presence of swarming stimuli was associated with an increase in insemination frequency from 74.3 to 97.7% in large cages. Transgenic males showed a lower competitiveness in large cages compared to small cages regardless of the presence of swarming stimuli.

**Conclusions:**

The results of the present study are discussed in view of the progressive evaluation of genetically modified *A.* *gambiae* strains and the potential applications of reproducing swarms in controlled conditions to dissect the mating behaviour of this species and the mechanisms controlling it.

## Background

Swarming by males is a natural mating behaviour of many Diptera [1, 2], including a major malaria vector mosquito, *Anopheles gambiae* sensu lato (*s.l*.) [3]. Swarms in Diptera are associated with overhanging trees, shrubs and contrasting-value ground markers [2], and among mosquitoes, swarms have been observed to occur during morning and evening twilight [1]. *Anopheles gambiae* swarms have been studied in the field, mainly in West Africa [4]. Males of this species specifically swarm near contrasting-shade ground features at evening twilight [4, 5], an activity lasting for approximately 30 min. Swarms are composed almost entirely of males into which females briefly fly and leave the swarm *in copula* [6]. Copulation lasts less than 20 s [6]. Swarms can contain up to thousands of males though tens to several hundred is more common [4, 7]. Field observations have produced the largest part of knowledge on mating behaviour of this species because *A. gambiae* swarms are difficult to obtain both in the laboratory and in large field cages [8], and such field work requires a great deal of experienced labour and long time periods to collect substantial data. The ability to reliably observe swarming in a controlled environment would thus be extremely valuable.

Mosquito insectaries typically provide light and dark periods to entrain behaviours including pupation, oviposition and mating, however wild anopheline mosquitoes brought into the laboratory do not mate at high rates making colonization difficult [9]. During the *A. gambiae* colonization process, environmental pressure acts on mating behaviour selecting stenogamy (in which mating takes place in restricted spaces like small cages) *versus* eurygamy (in which mating occurs in large spaces) [10]. Typical small laboratory cages (e.g. 30 cm per side) are not sufficiently large to permit swarming flights. There are few publications describing *A. gambiae* swarms indoors: Charlwood and Jones stimulated male swarming in a 1.7 m^3^ cage [11]; Marchand determined that provision of an artificial horizon that contrasted with a bright artificial sky encouraged swarming of *A.* *gambiae* and *Anopheles arabiensis* in a <1 m^3^ cage [9]. Others [12–15] developed a large cage called a “mesocosm” where they studied effects of sugar on mating performance of *A. gambiae*. In their setting, *A. gambiae* males were observed to swarm but those studies did not focus on swarm behaviour nor on the stimuli inducing it. Clear evidence of swarming activity effects on female insemination rates has not been published.

Although *A. gambiae* is one of the most studied mosquito species worldwide due to its role in malaria transmission, its mating behaviour and the mechanisms regulating it remain unclear. Little information is available on the male–female-environment interactions during mating [3, 4, 7, 16] and little is known of the molecular mechanisms that regulate these processes [17, 18].

Efforts to develop genetically-modified mosquitoes (GMM) aimed at reducing malaria transmission will benefit from understanding mating interactions and factors that are associated with mating competitiveness. Mating behaviour represents a key component for the transmission of selected transgenes to natural populations, and poor mating competitiveness or altered reproductive behaviour of GMM resulting from the rearing history or the transgene could cause genetic control strategies to fail [19]. Part of the reason for these gaps in knowledge of *A. gambiae* biology is that swarms are difficult to study in natural settings. Locating them can be challenging, and it is difficult to gather quantitative measurements. It is almost impossible to design, run and replicate experiments on *A. gambiae* swarms in the field.

In this study, venues designed to stimulate more natural *A. gambiae* mating behaviour in order to improve behavioural studies and evaluate the mating performances of transgenic males in the laboratory were created. Six large insectary cages (15.9 m^3^ each) were designed, modifying and expanding on the visual stimuli described by Marchand [9], to consistently induce swarming activity in *A. gambiae* males. Swarms were observed and their effect on the female insemination rate and male competitiveness of two transgenic male-sterile *A. gambiae* lines [20] was measured. For genetic control strategies that require male releases into the environment, mating competitiveness is critical for success. The goal of this study was to optimize the evaluation of the mating performance of transgenic males in the laboratory and to develop a laboratory experimental setting suitable to study mating behaviour of this species.

The results obtained are discussed in the perspective of the progressive evaluation of genetically modified *A. gambiae* lines. The ground-breaking research that could be produced by investigating male swarming behaviour in enclosed environments with the goal of expanding the basic knowledge of *A.* *gambiae* mating biology is also discussed.

## Methods

### Mosquito strains

The *Anopheles gambiae* G3 strain [21] was employed for swarm observations, while mating studies were performed with *A. gambiae* G3 *vs* two *A. gambiae* sterile male β2Ppo lines [20]. G3 was chosen because it is widely used as wild type strain to create genetically engineered mosquitoes, while the transgenic males employed belong to β2Ppo lines that are maintained by continuously crossing transgenic females to G3 males [20]. This produces pools of genetically similar transgenic individuals and non-transgenic “wild-type” comparators every generation that were competed for virgin female mates with either of two strains of transgenic males: β2Ppo1 and β2Ppo2 [20]. In heterozygous males of the β2Ppo lines, the expression of the I-PpoI enzyme in testes induces a strong bias toward Y chromosome–carrying spermatozoa and complete early dominant embryo lethality in crosses with wild-type females [20]. In both strains, I-PpoI is fused to green fluorescent protein (GFP), both of which are expressed in sperm.

### Cage design and environmental conditions

**Figure 1:**
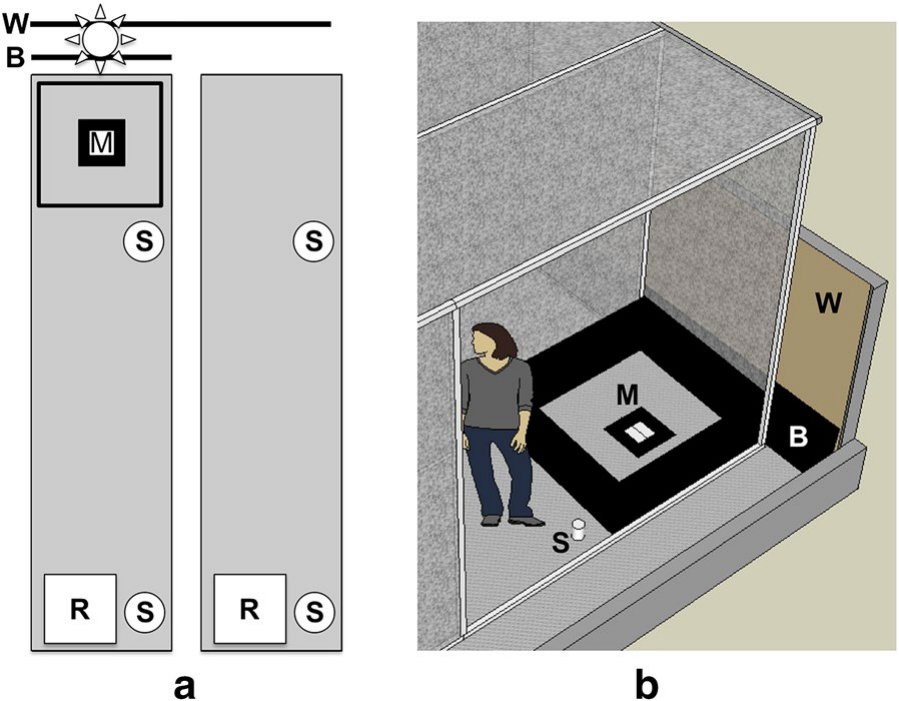
Illustration of the large cages. The typical arrangement of the swarming stimuli (*left*) is represented by the black-bordered swarm marker (*M*) surrounded by the black artificial horizon lining the interior of each cage. Above these and outside of the cage, the black baffle (*B*) is represented, preventing the three lights from shining directly into the cages but upward onto the wall (*W*). Also shown are the locations of sugar sources (*S*) and resting shelters (*R*) flat against the floor. The swarming stimuli and controls were switched between rooms to determine whether there was any effect of insectary.

The study was carried out in the insectaries of the Department of Experimental Medicine, Functional Genomics Center of the University of Perugia. A total of six experimental cages were built in two large chambers, *i.e.* Insectary Field 1 measuring 6.68 × 3.80 × 3.00 m (all dimensions are L × W × H), and Insectary Field 2 measuring 8.45 × 3.80 × 3.00 m (Figure 1). These studies were carried out in three types of cages: (1) 30 cm on each side white plastic Bugdorms (BioQuip Products, Inc., Rancho Dominguez, CA, US); (2) custom-made large cages without swarming stimuli; (3) custom-made large cages with swarming stimuli. Large cages (5.00 × 1.22 × 2.60 m each, Figure 1) consisted of white-painted wooden frames with walls and ceiling made of polyester mesh (1,290 µm openings, US size 15). Part of the front panel of each cage was made of a 50 × 125 cm Plexiglas sheet provided with two 20 cm square entrances that allowed access to cages through affixed sleeves opening to the chamber. Each large cage was provided with a terracotta resting shelter which was kept humid and two 500 ml cups containing a 10% sucrose solution, 0.1% methylparaben as preservative [22] and approximately 4 ml acacia honey as an attractant. White absorbent paper placed in the cup allowed mosquitoes to land and get the sugar meal without drowning. In each large cage, swarming stimuli (Figure 1), consisted of (1) square arena made of four black plastic sheets (Correx equivalent, 122 cm long, 50 cm high and 0.5 cm thick), located at the back of the cages; (ii) a contrasting ground mark consisting of a black plastic square (40 × 40 cm) and white plastic square (20 × 20 cm) centred on top of it in the centre of the arena; (3) a 2.700 K 8 W compact fluorescent light located on the floor at the back of the cage hidden from the mosquitoes by a black plastic Correx equivalent sheet (122 cm long, 50 cm high and 0.5 cm thick), diffusing light onto the back wall of the chamber simulating twilight (horizon lights). Temperature and relative humidity were kept stable during the studies *i.e.* 28.01°C SD ± 0.57°C and 72.1% SD ± 2.0%.

Light in the chambers was provided by four or five ceiling light fixtures in Insectary Field 1 and Insectary Field 2, respectively, producing a diffused and evenly distributed light. Each light fixture was equipped with three T5 80 W linear fluorescent bulbs with different emissions: 6.400, 4.000 and 2.700 K. Emitting temperature, day duration, dusk and dawn fading of ceiling lights, were controlled by Easy Color Control software (OSRAM SpA, Società Riunite OSRAM Edison Clerici, Milan, Italy).

Length of daytime was the same for the two large cage treatments but light locations and durations differed. In both treatments, dawn lasted for 30 m from dark to full light, and full light lasted for 11:30 h. In the chamber where cages lacked the swarming stimuli, sunset lasted for 1 h: 30 m of fading ceiling from full light to minimum emission and 30 m of 2.700 K tubes only at minimum emission. In the chamber where cages where provided with swarming stimuli, sunset lasted for 1 h: 30 m of fading ceiling light from full light to minimum power, overlapping with 60 m of twilight provided by the horizon light. Different combinations of visual stimuli described above were tested, and the presence/absence of swarming males was detected using binoculars (7x35 mm roof prisms Foton, CCCP).

### Design of competitive mating experiment

Assessing mating characteristics directly was facilitated by the fact that both strains have fluorescent sperm [20], which allowed us to determine whether wild-type, transgenic or neither type of male had mated females by examining their spermathecae. Because it is difficult to identify mixtures of fluorescent and wild-type sperm (the presence of fluorescent sperm is more easily concluded than mixtures), observations may bias the estimated frequency of matings in favour of transgenics. Only rarely (3 of 1,396 spermathecae) were we able to detect what appeared to be mixed matings. These cases were not included in the estimates of mating frequency. Larvae were reared using a diet reported previously [23]. Larvae were cultured according to a standard operating procedure (LV, personal communication). Two-day old virgin adults were introduced into three different type of cages: (1) Bugdorms; (2) large cages with visual stimuli inducing males to swarm; (3) large cages without visual stimuli. The mating competitiveness of the GMM lines was evaluated by introducing 25 β2Ppo1 or β2Ppo2 males, 25 G3 males, and 50 virgin G3 females in Bugdorms and 42 G3 males, 42 β2Ppo males and 84 G3 females in large cages. For details of the study results, see Table 1. Adults were collected from the cages after a week, females were dissected and spermathecae examined for the presence of sperm and fluorescence. Competitive mating with β2Ppo1 line was replicated by reversing the treatments in either chamber in order to determine whether there was any effect of environmental differences.

**Table 1:** Summary of competitive mating experiments employing males of β2Ppo1 line (above) and males of β2Ppo2 line (below)

Cage	**Treatment**	# of adults per cage β2Ppo1♂♂:G3♂♂:G3♀♀	(%) G3 ♂♂ survival	(%) β2Ppo1♂♂ survival	(%) ♀♀ survival	Insemination rate	(%) of ♀♀ inseminated by G3 ♂♂	(%) of ♀♀ inseminated by β2Ppo1♂♂
1	Bugdorm	25:25:50	53.6	46.4	76.0	73.7	75.0	25.0
2			50.0	50.0	86.0	60.5	69.2	30.8
3			54.1	45.9	82.0	75.6	71.0	29.0
A	Large Rep 1	42:42:84	–	–	54.8	65.2	96.7	3.3
B			–	–	69.0	75.9	95.5	4.5
C			–	–	72.6	76.7	97.8	4.3
D	Large swarm Rep 1	42:42:84	–	–	71.4	98.3	93.2	6.8
E			–	–	82.1	100.0	85.1	14.9
F			–	–	66.7	100.0	92.9	7.1
A	Large swarm Rep 2	42:42:84	59.5	50.0	82.1	97.1	94.0	7.5
B			60.0	40.0	76.2	96.9	95.2	4.8
C			52.4	11.9	63.1	94.3	94.0	6.0
D	Large Rep 2	42:42:84	78.6	50.0	78.6	74.2	81.6	18.4
E			71.4	11.9	77.4	61.5	97.5	2.5
F			61.9	35.7	73.8	83.3	89.8	10.2

**Cage**	**Treatment**	# of adults per cage β2Ppo2♂♂:G3♂♂:G3♀♀	(%) G3 ♂♂ survival	(%) β2Ppo2♂♂ survival	(%) ♀♀ survival	Insemination rate	(%) of ♀♀ inseminated by G3 ♂♂	(%) of ♀♀ inseminated by β2Ppo2♂♂
1	Bugdorm	25:25:50	46.9	53.1	88.4	81.6	45.2	54.8
2			52.4	47.6	90.0	86.7	69.2	30.8
3			48.7	51.3	94.0	74.5	54.3	45.7
A	Large	42:42:84	85.7	59.5	72.6	83.6	84.3	15.7
B			59.5	9.5	78.6	75.8	74.0	26.0
C			35.7	40.5	70.2	72.9	65.1	34.9
D	Large swarm	42:42:84	66.6	28.6	76.2	98.4	82.5	17.5
E			47.6	23.8	86.9	97.3	77.5	22.5
F			57.1	14.3	70.2	96.6	82.5	17.5

Cage number, treatment, number of adults introduced in each cage, WT and transgenic male survival, female survival, female insemination rate, and percentage of females inseminated by G3 and β2Ppo males are shown.

### Ethics statement

Adult females were fed using the Hemotek membrane feeding system (Discovery Workshops, Lancashire, England) and sterile cow blood (Allevamento Blood di Fiastra Maddalena, Teramo, Italy). The facility where the experiments were performed obtained the permit to host Class II genetically engineered organisms from the Italian Ministry of Health (permit no. PG/IC/OP2/13-002).

### Statistical analysis

All analyses were performed using R 3.0.1. In all cases, the data are proportions arising from counts of two conditions: (a) those females that mated and those that did not, (b) the number of females mated by wild-type and the number of females mated by GMM males, and (c) the number of females, wild-type males and GMM males at the start of the experiment and alive at the end. Each combination was bound as a single response variable enabling their assessment by factorial analysis of deviance. There was overdispersion in all data and a quasibinomial model fit was used. Non-significant model terms were eliminated, starting with which insectary was used and proceeding thereafter by stepwise deletion; deletion tests used “F” as appropriate to the overdispersion. Neither the date the experiment took place nor the individual cage code were included in the final analyses, but were checked for influence and excluded a priori. Male survival in Replicate 1 was not recorded.

## Results and discussion

### Stimulation of swarming behaviour

Matings were measured in two sizes of cages: three “small cages” in which no swarm stimuli were present and six custom “large cages” in which swarm stimuli were either present or absent. Mosquitoes in large cages were observed to swarm only when four visual stimuli were present and which were used for all “swarm stimuli” treatments (Figure 1): (1) fading ceiling lights that simulate dusk; (2) 50 cm high black panels arranged as a square box-like open-top arena (1.22 m on each side), to create a 360° artificial horizon; (3) a black and white landmark on the floor in the centre of the black arena; (4) an 8 W light located below the artificial horizon on one side of the cage (a “horizon light”) shining upward on the light-coloured wall to simulate a twilight sky. The last cue was important to stabilize the swarm above the landmark and to avoid males’ phototactic activity toward the ceiling lights which was observed in preliminary trials. In all respects, the swarms appeared similar to what is observed naturally. In the large cages, swarms of *A. gambiae* males were obtained with their centre positioned at the level of the horizon consistently above the landmark, only in the presence of all the visual stimuli described above. The percentage of swarming males of the total number of males present in the cages is variable ranging between 20 and 50%. Swarming behaviour started about 15 min after the beginning of the sunset beginning with 2–3 males. After that, more males joined the swarm that reached its maximum size when only the horizon lights were on, 30 min after the beginning of the twilight. During that time, the swarms had a sub-spherical shape, with their top slightly skewed toward the horizon lights. Its maximum size is about 60 cm wide and 50 cm height. In preliminary tests, the size of the cages (up to 3.00 × 2.50 × 2.50 cm, L × W × H), and the size of the black arenas (up to 3.00 × 2.50 × 1.50 cm, L × W × H) were increased. The swarm shape and size changed according to the cage setting maintaining almost the same width (about 60 cm) but increasing in height up to 1.00 m. In all cases, swarms were close to the cage floor with the lower males flying about 30 cm from the contrasting marker on the floor.

Routine laboratory culture selects strongly for stenogamy and it is surprising that selection has not eventually resulted in insemination of all females in small cages, particularly in a strain such as G3 that has been maintained in laboratories since 1975, approximately 730 generations. In preparatory tests, we used similar visual stimuli in small cages without obtaining an increase of mating frequency [67 and 58%, respectively with (4 replicates) and without (5 replicates) swarming cues, G test for goodness of fit, G = 0.089, p = 0.765]. Combined results demonstrated that although an increase in male flight activity and several mating events were observed at dusk in small cages, the characteristic male “dancing” activity associated with swarming at dusk was not observed. Flight activity of large numbers of males toward ceiling lights during the sunset phase was also observed in large cages without swarming stimuli, due apparently to male phototactic response, but it could not be described as swarm behaviour and no increase in female insemination rates was observed.

Results of mating competition experiments are summarized in Table 1.

### Adult survival and effect of swarming on proportions of females mated

Swarming is a behaviour that requires male flight for prolonged periods. Larger cages might demand greater flight performance of both males and females and affect adults’ ability to find shelter and sugar. The effects of swarming and cage size on male and female survival (after 7 days of cohabitation in all cases) for a wild-type comparator (G3 strain) and two strains of transgenic males [20] were determined.

#### G3 females

Considering all cages, neither the environmental chamber used nor the strain of males with which females cohabited affected survival (Table 2). However, wild-type female survival after 7 days was significantly higher in small cages (0.84 ± 0.03) than in large ones (0.76 ± 0.02). These explanatory variables, however, capture only 26% of the deviance in female survival. In large cages in which an effect of swarming could be detected, female survival was not influenced by either the strain of transgenic males with which they were housed (Table 2), by swarming, nor by their interaction. These factors explain only 5% of the deviance.

**Table 2:** Summary of the model deletion tests to identify effects on the survival of the *Anopheles gambiae*

	Female survival			Wild type male survival			GMM male survival		
	F	d.f.	p	F	d.f.	p	F	d.f.	p
All cages									
Insectary	0.05	14,15	0.82	1.37	14,15	0.26	0.67	14,15	0.43
Strain	0.78	13,14	0.39	0.25	15,16	0.63	0.02	15,16	0.88
Cage Size	*4.72*	*16,17*	*<0.05*	2.56	16,17	0.13	*15.81*	*16,17*	*<0.01*
Large cages only									
Strain:Swarm	0.18	8,9	0.68	0.25	8,9	0.63	0.67	8,9	0.44
Strain	0.33	10,11	0.57	0.34	9,10	0.57	0.19	9,10	0.67
Swarm	0.00	9,10	0.96	0.94	10,11	0.35	0.21	10,11	0.66

Statistically significant results are in italics.

#### G3 males

Overall, non-transgenic comparator males survived at a higher rate than transgenic males (0.65 ± 0.06 vs 0.45 ± 0.06, F_17,18_ = 27.1, p < 0.01). For wild type males, cage size, strain of transgenic male with which wild-type males were housed and environmental chamber captured 22% of the variance in survival considering all cages. Wild-type male survival did not vary as a function of strain with which they competed or cage size. In the large cages, the model accounted for 14% of the deviance in the data and was a poor fit. Wild-type male survival was not affected by swarming, the strain of transgenic male with which they were housed nor by their interaction.

#### Transgenic males

The full model, which considered transgenic male survival in all cages, accounted for 52% of the deviance. No difference between the survival of transgenic strain males was detected but survival was greater for both strains in the smaller cages (71 ± 0.05% vs 32 ± 0.04%) (Table 2). Within the large cages, the model was a very poor fit and explained only 10% of the deviance in survival. Neither strain nor swarming contributed significantly to variation in survival nor was survival affected by an interaction between strain and swarming.

#### Female insemination rate

**Figure 2:**
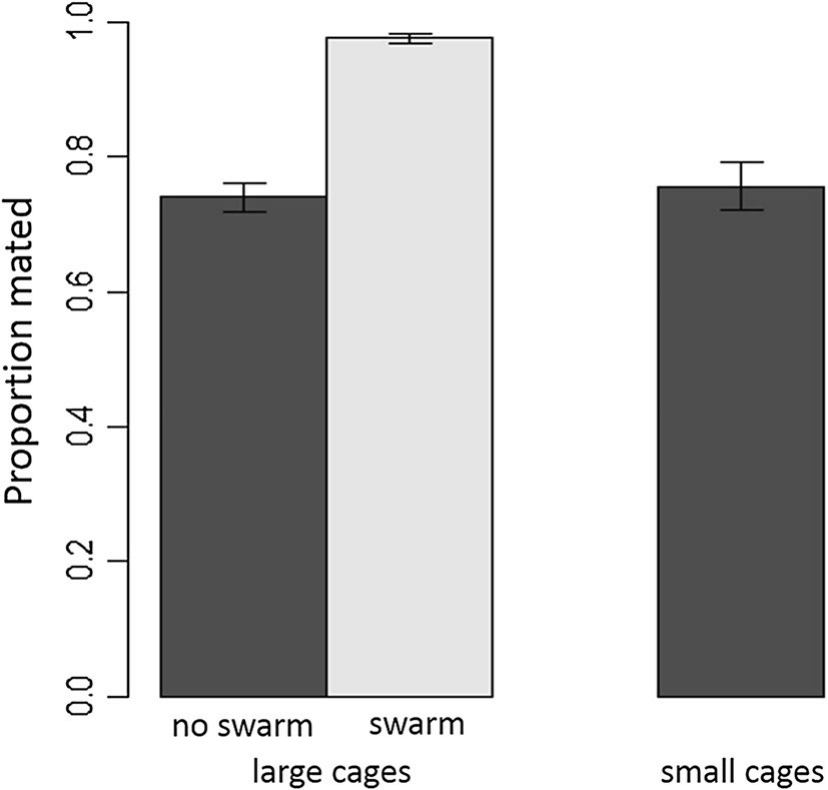
The total proportion of female *Anopheles gambiae* mated after 7 days as a function of swarm venue and cage size.

Only one explanatory variable affected the proportion of females mated and that was swarm venue presence in large cages (Figure 2). The proportion of matings was significantly higher in cages containing venues in which males had been observed to swarm (F_16,17_ = 136.45, p < 0.001). Neither strain of cohabiting transgenic male (F_15,16_ = 0.59, p = 0.45) nor its interaction with swarming (F_14,15_ = 0.28, p = 0.60) were significant contributors. Overall, neither the insectary, type of cohabiting male nor cage size affected the proportion of females mated (Table 3). The proportions of females mated did not differ depending on which transgenic strain of male was present nor was there an interaction between transgenic strain and swarm venue stimuli.

**Table 3:** Summary of the model deletion tests to identify effects on the mated status of female *Anopheles gambiae*

Both cage sizes	Proportion of females mated			Proportion of transgenic matings		
	F	d.f.	p	F	d.f.	p
Insectary	1.19	19,20	0.28	1.20	19,20	0.29
Strain	3.46	21,22	0.08	*27.44*	*21,22*	*<0.001*
Cage Size	0.03	20,21	0.87	*26.54*	*21,22*	*<0.001*
Swarm	*134.45*	*22,23*	*<0.001*	0.62	20,21	0.44

Statistically significant results are in italics.

The observation of male-swarming correlating with higher mating frequencies draws attention to the male’s role in mating and to the possible stimulation of female receptiveness by swarming activity. In *Aedes aegypti*, swarming males produce a volatile aggregation pheromone that attracts both males and females [24]. It is not known if the same happens in *A. gambiae* and these two species have a different swarming behaviour and mating strategy. The mechanism that results in difficulty in obtaining matings in the lab among field collected mosquitoes may be that many females will not mate except in a swarm. It remains difficult to know if male attempts at copulation are less frequent when they do not swarm or if some females are rejecting males that are attempting to copulate outside of a swarm formation. The propensity of at least a significant proportion of adults to mate in swarms has not been lost in these strains and we have shown in large cages that swarm behaviour increases insemination rate by approximately 24%. These data are consistent with the results of Dao et al. [25], which showed that *A. gambiae* probably has an alternative mating strategy such that not all mating occurs in swarms. In the field, Dao et al. calculated that mating in resting places where males do not swarm occurs at a low rate (between 6 and 15%), and it is plausible that this frequency increases during colonization in small cages, becoming predominant over mating in swarms after several generations in captivity, as detected in this study.

Female insemination rates obtained in large cages in other studies where swarms were observed [13, 14], were not as high as those we obtained. It is difficult to explain why but authors speculate that the visual stimuli produced, which were able to induce males to swarm, also have an effect on virgin females attracting them to the mating arena, a behaviour already reported [11].

### Competitiveness of transgenic males in the various settings

**Figure 3:**
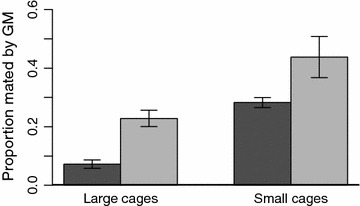
Transgenic male matings: The proportion of female *Anopheles gambiae* mated by β2Ppo1 (*dark bars*) and β2Ppo2 (*light bars*) when competing with wild-type males in large or small cages.

Short-term mating competitiveness experiments assess an important component of fitness and conducting them in large cages provides important preliminary information on the performance of GMM [26]. Considering only large cages in this study, neither the insectary used nor swarm stimuli affected the proportion of matings by transgenic males in large cages (Figure 3, Table 3). Nor did the ratio of transgenic to non-transgenic males surviving at the end of the experiment have any effect on the proportion of transgenic matings (F_15,16_ = 1.31, p = 0.27). In contrast, the two cage sizes had very different proportions of transgenic male matings: transgenic males competed more successfully in small cages vs. large ones. The strains also differed: β2Ppo2 achieved a higher proportion of matings than β2Ppo1 in all cases. The mating competitiveness estimates of “C”, based on the Fried index [27], of male β2Ppo1 and β2Ppo2, respectively in small cages were 0.396 (± 0.033) and 0.834 (± 0.222) and 0.081 (± 0.18) and 0.305 (± 0.051) in large cages.

**Figure 4:**
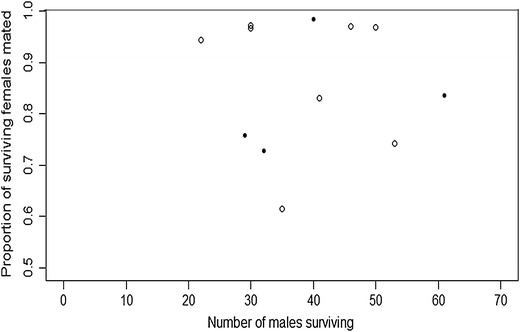
Proportion of inseminated females as a function of surviving males.

Unexpectedly, the proportion of females inseminated by transgenic males was not affected by transgenic male survival (Figure 4), being lower than that of non-transgenic males in large cages. This suggested that matings were completed early during the course of each experiment. In subsequent mating studies, it was observed that when large cages were populated with 2-day old virgin non-transgenic adults, 97.3 and 77.3% of females were inseminated in “swarm” and “no-swarm” treatments respectively, after only two nights. These values were similar after 5 and 7 nights in concurrent experiments. Therefore, *A. gambiae* male survival may not be a strong determinant of mating competitiveness in assays using cohorts of coetaneous adults. This finding should be taken into account for modelling purposes and allows mating competition experiments to be performed more quickly with *A. gambiae*, since they currently often take around 1 week [28]. The preparatory test described above indicate that most matings occur early during the course of the experiments and the insemination rate does not change over the subsequent 7 days. It has been shown that in different mosquito species, a single male can mate several females and males can replenish their sperm supply when depleted [29]. Therefore, it is reasonable to assume that the males that mated the majority of females within 2 days in the “no-swarm” treatments had a sufficient supply of sperm for mating the remainder within 7 days but did not. One might hypothesize that the mating limitation in the no-swarm treatments depends upon a female behaviour that is not stimulated under these conditions.

Independent studies indicated that competitiveness of these transgenic strains is consistent with the hypothesis that larger cages challenge mating ability more than small ones. The same transgenic strains tested here were competed previously in intermediate sized cages of 8.5 m^3^ [28]. Those cages did not contain any features identified here that might stimulate swarming (MQB personal communication). They obtained a similar proportion of inseminated females (75% after 7 days), as we observed here in the absence of swarming. The authors observed estimates of competitiveness intermediate to those determined here in smaller and larger cages: 0.116 (± 0.079) and 0.640 (± 0.036) for β2Ppo1 and β2Ppo2, respectively.

## Conclusions

The ability to reliably stimulate swarming in the laboratory in large cages provides both insights and an opportunity to study questions related to factors controlling assortative mating, perception and the evaluation of strains being considered for field release. The lack of stimuli for swarm behaviour, where most natural mating is believed to occur, and culture in small cages, mean that *A. gambiae* mating behaviour in insectaries is quite different from that which occurs in the field. Results from studies aimed at revealing factors affecting mating behaviour might only be a poor proxy for natural behaviour if males are in laboratory cages and do not have the possibility to swarm.

These data together demonstrate that for these strains, males that are observed to be inferior when competing for mates in small cages can be expected to have even less ability to compete in larger cages.

Previous research on *A. gambiae* swarms in the laboratory [9–11] provided a basis to study the natural mating behaviour of this species in enclosed environments. Although several studies describing *A. gambiae* mating behaviour in the field have been published since then [3, 4, 7, 16, 30, 31], parallel research in the insectary has not been developed. The main goals of swarm studies in the field have been to explain mating choice and reproductive isolation within the *A. gambiae* complex. Incipient speciation between the M and S molecular forms, now elevated to species rank [32], has been discussed [33], and there is evidence showing that gene flow between the molecular forms is reduced due to the presence of premating barriers. Reproducing M and S swarm segregation in the laboratory might provide the basis for revealing the mechanisms controlling assortative mating amongst them and will be a major component of our future activities. The behavioural processes leading to this reproductive isolation among wild populations are still not fully understood and seem to vary among populations and geographical areas [34]. The lack of a model that reproduces swarm behaviour in laboratory cages makes it difficult to investigate the mechanisms controlling the reproductive isolation within the *A.gambiae* complex. A starting point to reveal the mechanisms regulating non-random matings between M and S in the open field is to reproduce and study their mating behaviour in large insectary cages.

Results presented here are critical for achieving a more realistic evaluation of the mating performance, and fitness in general, of the GMM *A.gambiae* lines. The use of similar methods to reproduce *A.gambiae* swarms in experiments aimed at assessing the fitness cost of genetic modifications and more generally to study different aspects of mating behaviour of this species are strongly encouraged.
